# *Streptococcus mutans* carriage in the saliva of mothers and its association with dental caries and *Streptococcus mutans* carriage in the saliva of children between 6 and 30 months old in a low‐income setting in Karachi, Pakistan

**DOI:** 10.1002/cre2.648

**Published:** 2022-09-30

**Authors:** Ambreen Nizar, Maheen Sheikh, Farhan R. Khan, Najeeha Talat Iqbal, Syed I. Azam, Shahida Qureshi, Asad Ali, Fyezah Jehan

**Affiliations:** ^1^ Department of Pediatrics and Child Health Aga Khan University Karachi Pakistan; ^2^ Dentistry Section, Department of Surgery Aga Khan University Karachi Pakistan; ^3^ Department of Community Health Sciences Aga Khan University Karachi Pakistan

**Keywords:** bottle‐feeding, breastfeeding, dental caries, early childhood caries, saliva, *Streptococcus mutans*

## Abstract

**Background:**

Early childhood caries poses a significant health issue in children under 6 years old. It is determined that *Streptococcus mutans* is a primary etiological agent, likely to be transferred through maternal contact.

**Objectives:**

To determine the association of maternal *S. mutans* counts with *S. mutans* counts in their children between 6 and 30 months of age, and to determine the maternal and child DMFT (decayed, missing, and filled teeth) indices.

**Material and Methods:**

A community‐based cross‐sectional study was conducted in Karachi, Pakistan. A sample of 193 dyads of mother–children (6–30 months of age) was selected via purposive sampling. Saliva samples of the dyads were collected to assess *S. mutans* count. Caries assessment was performed for both using the DMFT index. A pretested questionnaire was used. The association of bottle‐feeding, oral hygiene measures, and other factors with *S. mutans* counts in children were also explored. Zero‐inflated negative binomial regression model at a 5% level of significance was applied using STATA version 12.0.

**Results:**

Out of 193 children, 109 (56.47%) were males and 84 (43.52%) were females. The mean age of mothers and children was 29.4 ± 6.2 years and 19.54 ± 6.8 months, respectively. Maternal *S. mutans* counts were not statistically associated with child's *S. mutans* counts (Mean child's *S. mutans* count ratio: 1; 95% confidence interval [CI]: 1, 1.01; *p* = .882). Compared with children who were breastfed, *S. mutans* counts were higher in children who were bottle‐fed (mean *S. mutans* count ratio= 4.85 [95% CI: 1.53, 15.41], *p* = .007). Age of mother and present caries status of mothers was significantly associated with the child's *S. mutans* count.

**Conclusion:**

No association between maternal *S. mutans* and child *S. mutans* was observed. However, maternal age, children who were breastfed, children who did not use pacifiers, and children with mothers who did not have caries, exhibited low *S. mutans* counts in their saliva.

## WHY THIS PAPER IS IMPORTANT

- No association was observed between maternal *S. mutans* and child *S. mutans* count.
- Breastfeeding, pacifier use, increasing maternal age, and absence of maternal caries were linked to lower *S. mutans* counts in children.

## 1 INTRODUCTION

Early childhood caries (ECC) is a significant public health problem prevalent in children under 6 years of age, with caries developing rapidly after the eruption (Xu et al., 2014). Children in high‐income countries are less likely to develop caries, with a 5%–20% prevalence in Europe (Saleem et al., 2015) and up to 45.8% in the United States between 2 and 19 years of age (Fleming & Afful, 2018). In lower‐ and middle‐ income countries such as South Asia, the prevalence can be as high as 46.9% in India (Ganesh et al., 2019), 23% in Sri Lanka (Shahim et al., 2004), and 50.1% in Pakistan (Charania et al., 2011). Most research on ECC is focused on preschool children between 3 and 6 years of age. However, children less than 3 years of age are also very susceptible to dental caries.

The proposed factors increasing vulnerability in children include an underdeveloped host defense system, hypoplastic defects on newly erupted tooth surfaces, transition from breast to bottle‐feeding, and subsequently weaning on solids at 6 months old (Lynch, 2013). The American Academy of Pediatric Dentistry records that children under 3 years of age may exhibit signs of smooth‐surface caries, which is indicative of severe ECC (S‐ECC).

While substantial data are present on the factors associated with caries in permanent teeth, there is a relative gap in knowledge in the realm of contributive factors to caries development in infants and young children (Prakash et al., 2012). Chief factors associated with the initiation and progression of dental caries include bacterial load in saliva and plaque, educational background, oral hygiene health awareness, and approaches implemented by caretakers (Avasare et al., 2017).

The mutans group of Streptococci is the principal etiological agents of ECC (Köhler et al., 1984), with *Streptococcus mutans* and *Streptococcus sobrinus* being the most common etiological agents. These organisms are nonmotile, catalase‐negative, facultative anaerobe, and gram‐positive cocci (Law et al., 2007). Longitudinal studies have found that the earlier the acquisition and colonization of *S. mutans*, the greater the incidence of caries at a later age (Berkowitz, 2006; Köhler et al., 1984). *S. mutans* has a weak capacity to become attached to epithelial surfaces, which correlates with studies, suggesting that *S. mutans* is not typically detected in predentate infants and colonization by this organism before teeth eruption is unlikely (Berkowitz, 2006). Recent studies, however, have proposed that bacteria can colonize the furrows of the tongue in predentate infants, serving as an ecological niche for colonization (Berkowitz, 2006). Numerous studies suggest that the initial colonization can vary between 7 and 36 months, coinciding with the eruption of primary teeth (Law et al., 2007). A systematic review reported that cohorts with *S. mutans* count >10⁵ cfu/ml showed a higher caries outcome. The results showed a 55% lower risk of caries in subjects with *S. mutans* counts <5 × 10⁵ cfu/ml (relative risk 0.45, 95% confidence interval [CI]: 0.34, 0.59, *p* < .00001) (Leal & Mickenautsch, 2010).

*Streptococcus mutans* is thought to be transferred during intimate contact between mother and child during the initial 2–3 years of life (Parisotto & Steiner‐Oliveira, 2010). The risk of ECC is high if *S. mutans* is acquired during the predentate stage (Ramos‐Gomez, 2012). This may indicate a correlation between high maternal and child salivary *S. mutans* counts (Law et al., 2007). Certain recent studies have also displayed statistically significant correlations between maternal decayed, missing, and filled teeth (DMFT) scores and *S. mutans* similarity levels in mothers and children (Esra et al., 2020).

Prior work on the subject suggests that ECC can be prevented if resources and strategies are directed to control “upstream“ determinants combined with early detection and intervention. To plan and implement such preventive programs in a given population, knowledge about the existing practices and attitudes related to oral health is essential. Studies on ECC in younger age groups below 3 years are lacking and it is recommended by many researchers that children under age 2 should be included to study the factors associated with S‐ECC, due to the susceptibility for ECC right after the eruption and before final maturation coinciding with the time when the child is breastfed. Previous research also employed small sample sizes, which could not determine the impact of all accounted variables on the *S. mutans* count. It is therefore imperative to study the factors associated with ECC in this age group, particularly factors and practices followed by mothers.

The prevention of ECC depends on the execution of early detection strategies, timely intervention, and oral health awareness for children (Gussy et al., 2006). Pertinent to Pakistan, existing literature details the prevalence of ECC in children between the ages of 3 and 5, along with a look at the association between oral hygiene and dietary factors with ECC (Charania et al., 2011). However, the novelty of the current study lies in the younger age group of 6–30 months targeted, due to the lack of information regarding this cohort (Iida et al., 2007).

Prior studies have specified the role of dietary factors and health practices in ECC, although with a small sample population and exclusion of related factors including breastfeeding (Agarwal et al., 2011). Thus, the hypothesis of the study states that children are more likely to have *S. mutans* presence if the mother is also positive for *S. mutans*, linking bacterial transmission to proximity and feeding practices. The primary objective of this study is to determine the association of maternal *S. mutans* carriage with *S. mutans* carriage among children between 6 and 30 months of age. The secondary objective is to determine the frequency of DMFT among the mothers and children in this cohort.

## 2 MATERIAL AND METHODS

### 2.1 Study setting, participants, and data collection

A cross‐sectional study was conducted from February 2015 to April 2015 in a peri‐urban setting of Rehri Goth, a settlement located in Bin Qasim Town, Karachi, the largest metropolitan hub of Pakistan. The Department of Pediatrics and Child Health at The Aga Khan University (AKU) maintains demographic surveillance of this area, where a primary health center provides free health care services for common childhood illnesses up to 5 years of age.

**Figure 1:**
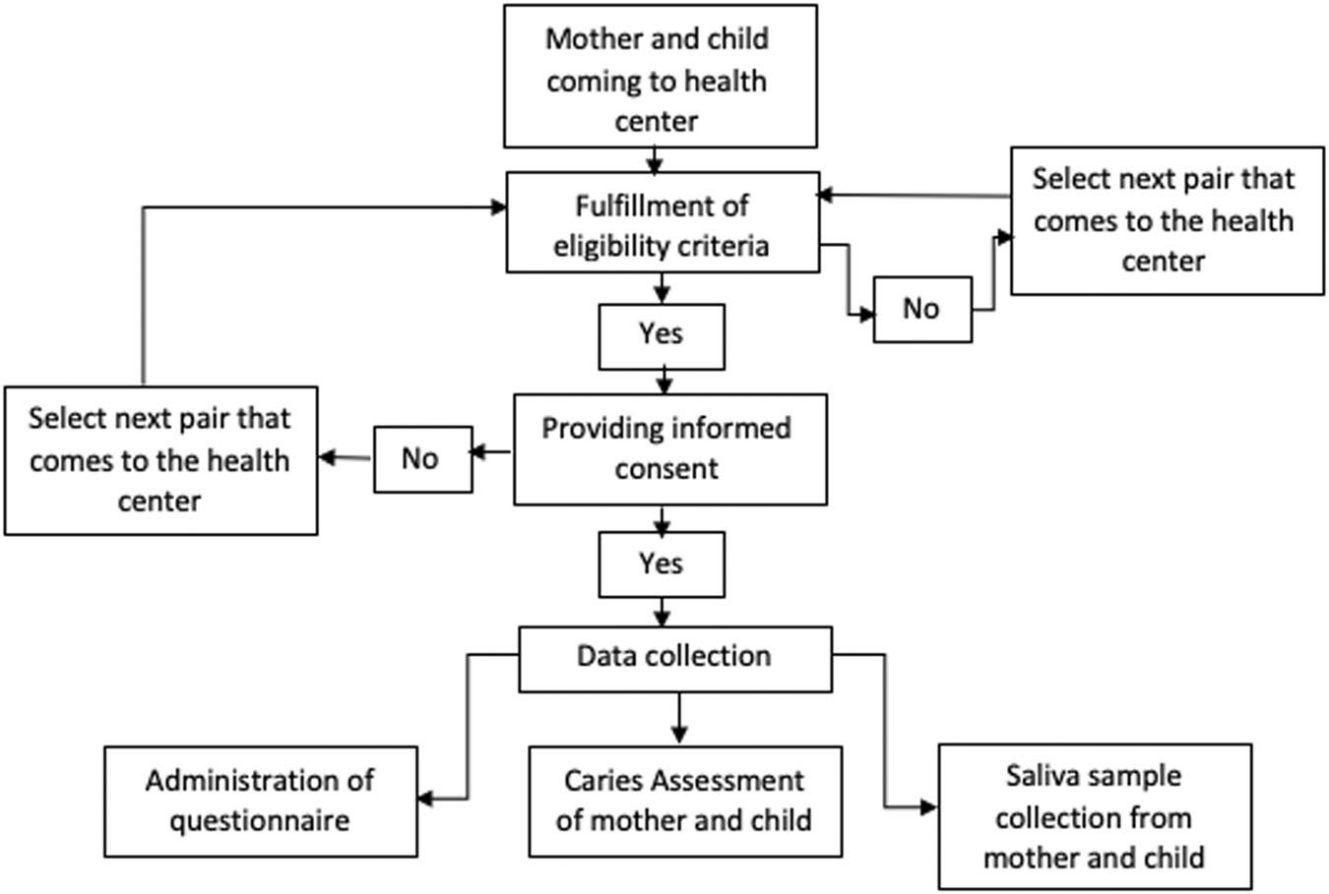
Study flow diagram

A basic screening form for the eligibility criteria is filled out for every mother–child pair coming to the health center (Figure 1). If the criteria are fulfilled, informed consent is taken and a study ID is assigned before the initiation of data collection.

### 2.2 Eligibility criteria

*Inclusion criteria for children*:

1. Age between 6 and 30 months old.
2. Mother is the primary caretaker of the child.
3. Child who was breastfed.
4. Children whose mother provided informed consent.
5. Child must have at least one tooth erupted in the oral cavity.

*Exclusion criteria for children*:

1. Taking any medicines affecting oral health (should not have received any antibiotics within the last 3 months).
2. Cleft lip and palate.
3. Any developmental disability associated with cleft lip/palate (e.g., Down syndrome).

*Inclusion criteria for mothers*:

1. Mother is the primary caretaker of the child.
2. Minimum of 20 teeth present.
3. No dental prosthesis.
4. Mother who provided informed consent.

*Exclusion criteria for mothers*:

1. Taking any medicines affecting oral health (should not have received any antibiotics within the last 3 months).

Nonprobability purposive sampling was done by selecting mother–child pairs that presented to the health center and fulfilled the eligibility criteria. A total sample of 193 mother–child pairs were enrolled. Data were collected on demographics, feeding practices, oral hygiene information, mouth‐breathing status of the child, and assessment of caries through DMFT scoring. A saliva sample was collected.

Written informed consent was taken after which information about birth weight, demographics, breastfeeding, and oral hygiene was recorded. Information was recorded through a questionnaire approved by the departmental and institutional review boards. DMFT scoring and data recording were conducted exclusively by a trained dentist on site, with frequent visits to the study site by the primary team for quality assurance measures. For children, the corresponding standard decayed, missing, and filled (primary) teeth (dmft) index was used.

### 2.3 Sample collection, transport, and processing

#### 2.3.1 Saliva sample collection

The saliva samples were collected from the mothers in a disposable plastic container containing 2 ml of autoclaved liquid Amies transport medium, a charcoal‐containing suspension used for the transport of the collected samples. Approximately 4 ml of passive drool was obtained because it is both cost‐effective and approved for use with all analytes. Whole saliva is the gold standard when collecting oral fluid for biological testing as it avoids localized secretions of specific salivary glands, making it a more consistent specimen. The time duration for collection of saliva ranged from 5−10 min.

For children, the saliva samples were collected in Oracol swab and storage tubes, a noninvasive method for collection of oral fluid, with 1 ml of autoclaved liquid Amies transport medium. Oracol swabs are manufactured in longer lengths and narrow widths to allow one end of the swab to be held by a parent or technician while the other end is placed in the child's mouth. It is durable and can withstand chewing, making it appropriate for use in children. Saliva collection is completed until the lower‐third of the swab is saturated, typically taking between 60 and 90 s. The volumes of salivary samples recovered ranged from 200 to 1000 µl.

#### 2.3.2 Precautions required

It was recommended that study participants should not have smoked, eaten, consumed any liquids, brushed their teeth, or used mouthwash within 1 h of the appointment.

#### 2.3.3 Sample processing

The samples were transferred at 4°C to maintain the viability of the microorganisms and prevent the overgrowth of commensals. All the samples were transported to the Infectious Disease Research Laboratory (IDRL) at AKU for processing. Once at the lab, the samples were serially diluted at 10^2^, 10^4^, and 10^6^ in phosphate‐buffered saline (PBS) and plated on Mitis Salivarious Bacitracin (MSB) agar. A total of 0.1 ml of respective dilution was plated on the MSB agar plate and a spreader was used to distribute the inoculum. After 48−72h, the number of colony forming units (CFU) was counted and reported. Subsequent confirmation of *S. mutans* growth was conducted by biochemical tests, including catalase and esculin tests, and analytical profile index  strips.

### 2.4 Statistical analysis

The sample size was calculated through NCSS‐PASS (Power Analysis and Sample Size) 2008. A total of 193 mother–child pairs were required to achieve 80% power with an anticipated correlation of more than 0.2 and a level of significance of 5%.

The statistical analysis of the data was performed using STATA version 12. Zero‐inflated negative binomial (ZINB) regression was used to observe the association of *S. mutans* count in the mothers and children. Variables with a *p* value of less than .25 and biological plausibility were considered for the multivariable analysis. A purposeful selection of participants was used for building the main effect model. Model appropriateness was assessed using the Vuong test (Ha: ZINB is a better model than the standard negative binomial model), which rejected the null hypothesis at a 5% level of significance (*p* value = <.001), concluding that the ZINB model was superior to the standard negative binomial model.

## 3 RESULTS

Overall, 193 mother and child dyads were studied. The mean ages of the mothers and children were 29.4 ± 6.2 (SD) years and 19.54 ± 6.8 months, respectively. Roughly 55% of the mothers had received no formal schooling. *Streptococcus mutans* was detected in the salivary samples of 43.5% (84) of the children, and 18.7% (36) of the mothers (see Table 1).

**Table 1:** Demographic information and distribution of *S* ***treptococcus*** *mutans* count

For child			For mother		
Demographic information of the child	*n*	%	Demographic information of the mother	*n*	%
Male	109	56.5	Formal education	86	44.6
Female	84	43.5	No formal education	107	55.4
Age of the child (months)			Age of the mother (years)		
< 12	24	12.4	<25	42	21.8
12–17	42	21.8	25–34	94	48.7
18–23	46	23.8	>35	57	29.5
24 and above	81	42			
Mean age of the child in months (SD)	19.54 (6.67)		Mean age of the mother in years (SD)	29.4 (6.2)	
Bacterial colonization			Bacterial colonization		
>0 CFU/ml	84	43.5	>0 CFU/ml	36	18.7
=0 CFU/ml	109	56.5	=0 CFU/ml	157	81.3
Median salivary bacterial counts (IQR)^a^	62,500 (13,500‐400,000)		220,000 (33,500–755,000)		

Abbreviation: CFU, colony forming units.

^a^ Median and interquartile ranges (IQR) are reported for those who have bacterial counts >0 CFU/ml only.

Nearly 96.9% of mothers reported past or current exclusive breastfeeding, with 53% of mothers having exclusively breastfed for at least 6 months. Approximately 53.8% of children were bottle‐fed. Around 5.7% of mothers reported that they either had used or were using a pacifier. Only 2.1% of mothers reported that they started cleaning their child's teeth when they were less than 6 months old. A total of 17.1% of mothers reported using a brush to clean their child's teeth. A total of 58.5% of mothers cleaned their child's teeth once daily (see Table 2).

**Table 2:** Descriptive analysis of feeding practices and oral hygiene measures

Variable	Categories	*N*	%
Exclusive breastfeeding	Yes	187	96.9
	No	6	3.1
Duration of exclusive breastfeeding in months	Less than 6 months	88	47.1
	6 months	61	32.6
	More than 6 months	38	20.3
Months of exclusive breastfeeding mean (SD)	5 (3.1)		
Frequency of breastfeeding during exclusive breastfeeding	Less than 5 times	2	1.1
	5–10 times	181	96.7
	More than 10 times	4	2.1
Start of complementary feed	Below 6 months	13	6.9
	At 6 months	14	7.5
	Above 6 and below 12 months	120	63.8
	At or above 12 months	41	21.8
Mean age of start of complementary feeding in months (SD)	8.9 (2.9)		
Breastfeeding during complementary feeding	Yes	162	86.1
	No	26	13.8
Use of bottle‐feeding	Yes	104	53.8
	No	89	46.1
Duration of the bottle‐feeding mean (SD)	11.04 (6.70)		
Use of pacifier	Yes	11	5.7
	No	182	94.3
Age at which cleaning of teeth was started	Before 6 months	4	2.1
	6‐12 months	20	10.4
	After 12 months	17	8.8
	Never cleaned child's teeth	152	78.7
Method of cleaning	Brush (manual/electric)	7	17.1
	Finger	10	24.4
	Miswak or Dandasa	2	4.8
	Soft clean cloth/gauze	17	41.4
	Rinsing with water	5	12.2
Frequency of child's teeth cleaning	Once a day	24	58.5
	Twice a day or more	12	29.3
	Very rare (once or twice a week)	5	12.2
Cleaning child's oral cavity after breastfeeding	Yes	26	13.5
	No	167	86.5
Mouth‐breathing (child)	Yes	55	28.5
	No	138	71.5

Children with carious teeth (i.e., having a dmft > 0) were 12 (6.21%) and mothers (DMFT > 0) were 130 (67.35%). Among children with ECC, none had received prior treatment. The maximum DMFT score for mothers was 26, while the maximum dmft score for children was 15. For both, the decayed component contributed the greatest to the DMFT score (see Table 3).

**Table 3:** Descriptive analysis of oral examination

Oral examination of child			Oral examination of mother		
Variable	Categories	*n*(%)	Variable	Categories	*n* (%)
Decayed teeth (child) “d”	Yes (*d* ≥ 1)	12 (6.2)	Decayed teeth (mother) “D”	Yes (*D* ≥ 1)	122 (63.2)
	No (*d* = 0)	181 (93.8)		No (*D* = 0)	71 (36.8)
Median number of decayed teeth in child's mouth (IQR)	4 (2.5–4.5)		Median number of decayed teeth in mother's mouth (IQR)*	3 (1–5)	
			Missing teeth in mother's mouth “M”	Yes (M ≥ 1)	54 (28)
				No (M = 0)	139 (72)
			Median number of missing teeth in mother's mouth (IQR)^a^	2 (1 – 3)	
			Filled teeth in mother's mouth “F”	Yes (F ≥ 1)	1 (0.5)
				No (F = 0)	192 (99.5)
Child's caries status based on “dmft” score	Carious (dmft ≥ 1)	12 (6.2)	Mother's caries status based on “DMFT” score	Carious (DMFT ≥ 1)	130 (67.4)
	Caries free (dmft = 0)	181 (93.8)		Caries free (DMFT = 0)	63 (32.6)
Median dmft score of the child (IQR)^a^	4 (2.5–4.5)		Median DMFT score of the mother (IQR)^a^	3 (2–6)	

Abbreviations: DMFT, decayed, missing, and filled teeth in permanent dentition; dmft, decayed, missing and filled teeth in primary dentition.

^a^ Median and Inter quartile ranges (IQR) have been reported for those who have scores ≥ 1.

3.1

Via the univariate stage of the ZINB regression model, it was found that the *S. mutans* count of the mothers was not associated with *S. mutans* count of the children (beta coefficient −3.42e−06, *p* value 0.40, confidence intervals −0.0000114, 4.54e−06). Likewise, the final model did not show any significant association between the mothers' and children's *S. mutans* count. The final model did not show a significant association between the maternal count with child count using adjusted β coefficients and robust standard errors (see Table 4). In the final model, the children's *S. mutans* count displayed a significant association with bottle‐feeding (*S. mutans* count ratio 4.85 [95% CI: 1.53, 15.41], *p* value = .007), pacifier use (9.29 [1.25, 68.71], *p* = .02), and mouth breathing (3.13 [95% CI: 1.17, 8.41], *p* = .023). The age of the mother was found to be weakly associated with an increase in *S. mutans* counts (1.07 [95% CI: 0.99, 1.16], *p* = .07). The caries status of the mother was also significantly associated with the child's *S. mutans* count (5.7 [95% CI: 1.51, 21.54], *p* = .01). Interestingly, no breastfeeding during complementary feeding was found to be associated with an increase in the child's *S. mutans* count (0.21 [95% CI: 0.06, 0.674], *p* = .009). The mean *S. mutans* count ratio for children who never had their teeth cleaned versus children whose teeth were cleaned starting between 6 and 12 months old was 3.78 ([95% CI: 1.17, 12.30], *p* = .026).

**Table 4:** Factors associated with *Streptococcus mutans* count in children 6−30 months old

Variables	Adjusted β coefficients (robust SE)	Unadjusted mean *S. mutans* count ratio	Adjusted mean *S. mutans* count ratio (95% CI), *p* values
Bottle‐feeding			
Yes	1.58 (0.59)	36.23	4.85 (1.53, 15.41), *p* = .007
No (Ref)		1	
Pacifier use			
Yes	2.23 (1.02)	102.51	9.29 (1.25, 68.71), *p* = .02
No (Ref)		1	
Mouth‐breathing (child)			
Yes	1.14 (0.50)	62.17	3.13 (1.17, 8.41), *p* = .023
No (Ref)		1	
Age of mother (in years)	0.07 (0.04)	1.28	1.07 (0.99, 1.16), *p* = .07
Caries Mothers based on “DMFT” score		1	
Caries free (DMFT = 0) (Ref)		1	5.7 (1.51, 21.54), *p* = .01
Carious (DMFT ≥ 1)	1.74 (0.68)	24.53	
Breastfeeding during complementary feeding			
Yes (Ref)		1	0.21 (0.06, 0.674), *p* = .009
No	−1.57 (0.60)	0.04	
Age at which teeth cleaning was started			
<6 months or >12 months	1.69 (0.57)	0.571	5.42 (1.77, 6.61), *p* = .003
6−12 months (Ref)		1	
Never cleaned the child's teeth	1.33 (0.60)	8.25	3.78 (1.17, 12.30), *p* = .026

Abbreviation: DMFT, decayed, missing, and filled teeth in permanent dentition.

## 4 DISCUSSION

This study displays no significant association between maternal and child *S. mutans* oral count, differing from prior literature suggesting a positive association although in older children (Priyadarshini et al., 2017). Uniquely we looked at children between 6 and 30 months of age to assess a correlation due to mother–child contact, rather than the influence of common dietary habits. Studying this age group was important because effective preventive measures can be implemented in this age group if pathology is identified. Existing studies have focused on the role of communal family dietary habits and oral hygiene, which may play a confounding role, making it difficult to solely attribute the maternal presence of bacteria to a child's oral bacterial colonization.

The total number of children from the study sample with carious teeth (i.e., dmft > 0) was 12 (6.21%) (see Table 5). Agarwal et al. *S. mutans* detection in 36.6% of children from 6 to 24 months of age, in comparison to a 43.5% prevalence among 6‐ to 30‐month‐old children in our study (Priyadarshini et al., 2017). Another study detailed that *S. mutans* was identified in 49% of the samples of children between 15 and 35 months old (Singh & King, 2003).

**Table 5:** Child *Streptococcus mutans* count by age

	Presence of *Streptococcus mutans* in children		Child caries status (dmft > 0)		
Child age (in months)	Yes	No	Yes	No	Total
<12 months	12	12	1	23	24
12–17 months	20	22	0	42	42
18–23 months	15	31	1	45	46
≥24 months	37	44	10	71	81
Total	109	84	12	181	193

Abbreviation: dmft, decayed, missing and filled teeth in primary dentition.

In the current cohort, *S. mutans* was detected in 18.6% (*n* = 36) of the mothers. Of these, 80.5% (*n* = 29) had caries. On the other hand, in the absence of *S. mutans* (80.4% (*n* = 157)), around 64% (*n* = 101) of mothers had caries (*p‐*value: .061) (see Table 6). This insinuates the presence of other organisms expediating the initiation and progression of caries. Aas et al. concluded through molecular methods that *S. mutans* is one of many other organisms leading to dental caries (Aas et al., 2008). The study also revealed that *S. mutans* was not detected in 10% of the subjects with dental caries. In this study, in contrast, *S. mutans* was not detected in 77% of mothers and 50% of children, proposing the need for further longitudinal studies to ascertain the spectrum of etiological agents.

**Table 6:** Mother and child's *Streptococcus mutans* and caries status

*Maternal Streptococcus mutans and caries status (n= 193)*			
*S. mutans* positive	38 (18.6%)	Presence of caries (DMFT > 0)	29 (80.5%)
		Absence of caries (DMFT < 0)	7 (19.4%)
*S. mutans* negative	157 (81.3%)	Presence of caries (DMFT > 0)	101 (64.3%)
		Absence of caries (DMFT < 0)	56 (35.6%)
*Child S. mutans count and caries status (n = 193)*			
*S. mutans* positive	84 (43.5%)	Presence of caries (dmft > 0)	6 (7.1%)
		Absence of caries (dmft < 0)	78 (92.9%)
*S. mutans* negative	109 (56.5%)	Presence of caries (dmft > 0)	6 (5.8%)
		Absence of caries (dmft < 0)	101 (94.4%)

Abbreviations: DMFT, decayed, missing, and filled teeth in permanent dentition; dmft, decayed, missing and filled teeth in primary dentition.

Our results showed an increase in the child's bacterial counts with an increase in the mother's age (child's *S. mutans* count ratio: 1.07, 95% CI: 0.99, 1.16, *p* = .07). Information collected on parity may be useful to tally the number of siblings with possible oral hygiene neglect. A positive association between breastfeeding during complementary feeding and a child's *S. mutans* count was found. In comparison, children who were not breastfed during complementary feeding had decreased *S. mutans* counts (*S. mutans* count ratio: 0.21, 95% CI: 0.06, 0.67, *p* = .009). This suggests a link between breastfeeding with higher *S. mutans* count, and children who are breastfed during complementary feeding are likely to have higher bacterial counts. On the other hand, children who are weaned off breast milk and onto other food sources are likely to have lower bacterial counts, posing the question of the role of prolonged breastfeeding and skin contact in *S. mutans* status. Previous literature on the subject also concurs with these findings, with a study showing that prolonged breastfeeding is associated with higher transmission and a higher caries rate (Agarwal et al., 2011). However, other studies have shown that breastfeeding may be linked with a lower *S. mutans* count (Priyadarshini et al., 2017), suggesting that the relationship between feeding practices and bacterial oral colonization may be multifactorial, including maternal bacterial status and race specificity. Breast milk contains high concentrations of secretory immunoglobulin A (IgA) antibodies, aiding in strengthening the oral defense system against pathogens (Baweja et al., 2017). IgA and IgG also have the capability to restrict streptococcal growth. Certain constituents of breast milk, including lactoferrin, lysozyme, albumin, and peroxidase have bactericidal activity against *S. mutans* and *S. sorbinusz*(Grenby et al., 2001).

Our study shows a positive relationship between bottle‐feeding, pacifier use, and mouth‐breathing with *S. mutans* count in children (*S. mutans* count ratio: 4.85, 95% CI: 1.53, 15.41, *p* = .007; 9.29, 95% CI: 1.25, 68.71, *p* = .02; and 3.13, 95% CI: 1.17, 8.41, *p* = .023, respectively). In this study, the use of pacifiers has been shown to be associated with increased counts of child's *S. mutans* (*S. mutans* count ratio: 9.29, 95% CI: 1.25, 68.71, *p* = .02), with similar findings being reported in Turkish and Japanese children (Ersin et al., 2006). Children who have never had their teeth cleaned had increased *S. mutans* count compared with those children who had their teeth cleaned starting from 6 to 12 months of age (child's *S. mutans* counts: 3.78, 95% CI: 1.17, 12.30, *p* = .003). It is recommended that children's teeth should be cleaned starting once the first deciduous teeth erupt. The oral cavity should be cleaned every time the child is breastfed or bottle‐fed. In the current cohort, 13.5% of mothers reported cleaning their child's oral cavity right after breastfeeding. This proportion is in accordance with the 11% reported in a study conducted in Fiji by Singh and King (2003). Experiments done by Weiss and Bibby found that if the surface of tooth enamel is treated with milk and then washed, the solubility of enamel is reduced by more than 20% (Singh & King, 2003).

The relationship between the detection of bacteria and the presence of caries is very well documented in adults, but not so much in children (Ramamurthy et al., 2014). In our study, the mothers' caries count was significantly associated with the child's bacterial count (child *S. mutans* count ratio 5.7, 95% CI: 1.51, 21.54, *p* = .01), which suggests mother‐to‐child transmission of bacteria.

Our study is the first conducted in South Asia looking at individuals between 6 and 30 months of age. Data collectors were blinded to the presence of bacteria at the time of caries examination. However, salivary samples were only collected at one time point, which may not reflect the dynamic changes that may happen with time; ideally, multiple samples should have been obtained on different days for a more reliable picture. Recall bias might have misclassified the feeding practices, while selection bias also posed a hurdle as only those mother–child pairs who presented to the health center were recruited. Measurement bias may have played a role in the collection of information as birth weight data were entirely based on the mother's recall while filling the questionnaire. Fundamentally, the development of caries is a time‐dependent process, which may be the cause of frank cavitation not being reflected in this young age group as it would in older children. Additionally, the bacterial strains involved could have been isolated for genetic analysis to confirm maternal‐to‐child transfer. Based on previous literature, potential confounders that could have played a role in the results and analysis include birth weight, maternal education, and family income. Since maternal education and family income do not themselves cause any pathology, they act as proxy measures for oral health and feeding practices, deeming them surrogate confounders.

Future implications of this study lie in maternal education of implementation of proper feeding practices. The use of pacifiers and excessive bottle feeding practices should be discouraged as they encourage oral bacterial colonization. Longitudinal studies have suggested that early acquisition of these bacteria may induce the development of immunoglobulins, providing an interesting platform to explore the significance of early bacterial colonization and subsequent caries incidence.

While no significant association between maternal *S. mutans* and child *S. mutans* was observed, it will be relevant to follow the *S. mutans* positive subjects to note any significant changes in caries incidence with progressing age.

### 4.1 Ethical considerations

Mild carious lesions were treated with atraumatic restorative treatment. Sealants were placed where required and oral hygiene education was given at the health center. Moderate to severe carious lesions were treated at the AKUH dental clinic.

## AUTHOR CONTRIBUTIONS

Ambreen Nizar, Maheen Sheikh, and Fyezah Jehan wrote the manuscript. Farhan Raza Khan and Asad Ali were involved in the conception of the project. Najeeha Talat Iqbal and Shahida Qureshi worked on the acquisition of data. Syed Iqbal Azam contributed to the analysis and interpretation of the results. All authors reviewed the manuscript.

## CONFLICT OF INTEREST

The authors declare no conflict of interest.

## ETHICS STATEMENT

The experimental protocol was approved by Aga Khan University's Ethical Review Committee. The collection and management of all biological samples were approved by the Ethical Review Committee and followed standard institutional practices. Written informed consent was taken from all participants.

## Data Availability

The datasets generated and analyzed during the current study are available in the Figshare repository, https://figshare.com/s/f218d5dfd5173ddd76e8.
